# Comparison of intravenous and topical tranexamic acid in total knee arthroplasty

**DOI:** 10.1186/s12891-018-2122-7

**Published:** 2018-06-13

**Authors:** Wenbo Wei, Shajie Dang, Dapeng Duan, Ling Wei

**Affiliations:** 1 Department of Orthopedics, Shaanxi Province People Hospital, Xi’an, 710004 China; 2 Xi’an JiaoTong University, Xi’an, 710004 China; 3 Department of Anesthesiology, Shaanxi Provincial Cancer Hospital, Xi’an, 710001 China; 4 Department of Pain, YangLing Demonstration Zone Hospital, No.15 Kangle street, Yang ling, Xi’an, 712100 China

**Keywords:** Total knee arthroplasty, Tranexamic acid, Hemoglobin drop, Intravenous injection, Topical injection

## Abstract

**Background:**

To investigate the clinical effectiveness of intravenous (IV) and topical tranexamic acid (TXA) in patients undergoing total knee arthroplasty (TKA) by comparing safety, efficacy and patient-reported outcomes.

**Methods:**

In this prospective single-blind clinical trial, 64 patients were randomized into two groups (*n* = 32 each). The Intravenous Group was administered TXA 10 mg/kg IV (Reyong, Shandong, China) 10 min prior to tourniquet deflation. In the Topical Group, 1.0 g TXA diluted in 50 ml of normal saline was injected into the surgical site, which was bathed in the solution for at least 5 min prior to tourniquet deflation. Outcomes included changes in hemoglobin levels, intra-operative, post-operative, and total blood loss, number of transfusions and number of transfused units, patient-reported postoperative Visual Analog Scale (VAS) score for knee pain, and complications.

**Results:**

There were no significant differences in intra-operative blood loss, post-operative blood loss, total blood loss, or post-operative decrease in hemoglobin in the Intravenous Group versus the Topical Group. The number of transfused red blood cell units was significantly greater and-post-operative VAS score was significantly lower in the Intravenous Group. There were no differences in post-operative thromboembolic complications between groups.

**Conclusions:**

Topical TXA is not inferior to IV administration in reducing perioperative blood loss in primary TKA. However, the influence of injection volume of locally applied TXA on post-operative knee pain warrants further investigation.

**Trial registration:**

Clinical ethics committee of Shaanxi People’s Hospital (2009), No.125. (ChiCTR 1,800,015,793) registered on 20/04/2018.

## Background

Since the 1960s, total knee arthroplasty (TKA) has become one of the most commonly performed orthopedic operations for the treatment of end-stage knee arthritis. Although TKA provides substantial pain relief and greatly improves orthopedic patients’ quality of life, the surgery is associated with complications; in particular, patients undergoing TKA are at risk for bleeding and may require transfusion. As transfusions extend patients’ rehabilitation time, prolong length of hospital stay, and represent a considerable cost [1–5], control of bleeding using antifibrinolytic agents such as tranexamic acid (TXA) is a primary focus in TKA [6–10].

TXA exerts its antifibrinolytic effects by inhibiting plasminogen, which prevents plasminogen activation and the binding of plasmin to fibrin. This leads to clot stabilization and decreases blood loss in surgical patients [11].

Previous publications have reported on the safety and efficacy of intravenous (IV) TXA for TKA. IV TXA is associated with decreased intra-operative blood loss, transfusion rates, and drop in post-operative hemoglobin concentrations, with no increase in the incidence of post-operative infections or thromboembolic events compared to placebo in patients undergoing orthopedic surgery [12–16]. However, patients with medical conditions such as renal insufficiency, history of previous deep vein thrombosis (DVT), and cerebrovascular and cardiac disease [17] may not tolerate intraoperative IV TXA; topical administration of TXA maybe more appropriate for these patients. Evidence suggests that topical and IV TXA have similar safety and efficacy in TKA; however, there is no consensus regarding the most clinically effective regimen for TXA administration. Clinical effectiveness is determined by the therapeutic benefit and adverse effects of a therapeutic approach, as evaluated by patients and clinicians. To the authors’ knowledge, no studies comparing different regimens of TXA administration in TKA include patient-reported outcomes, such as patient-reported knee pain, which may have a substantial impact on length of hospital stay and delay rehabilitation, resulting in increased cost associated with surgery. Therefore, the purpose of this study was to investigate the clinical effectiveness of IV and topical TXA in patients undergoing TKA by comparing safety, efficacy, and patient reported outcomes represented by the postoperative Visual Analog Scale (VAS) score for knee pain.

## Methods

This prospective single-blind randomized controlled trial included patients with knee osteoarthritis undergoing unilateral primary TKA performed by senior surgeons at the Department of Orthopedic Surgery, Shannxi Provincial People’s Hospital between March, 2010 and October, 2013. The study protocol was approved by the institutional review board (Clinical ethics committee of Shaanxi People’s Hospital, Shaanxi, China), and all study participants provided written informed consent.

Inclusion criteria were 1) ≥18 years old; 2) American Society of Anesthesiologists (ASA) score ≤ 3; and 3) planned TKA due to degenerative arthritis of the knee. Exclusion criteria were 1) cardiovascular problems (e.g., myocardial infarction, atrial fibrillation, angina); 2) cerebrovascular conditions (e.g., previous stroke or previous vascular surgery); 3) thromboembolic disorders; and 4) renal insufficiency.

On the day of surgery, included patients were randomized 1:1 into two groups using a random number table and closed envelopes (*n* = 32 each group). The Intravenous Group was administered TXA 10 mg/kg IV (Reyong, Shandong, China) 10 min after placement of a loose tourniquet. In the topical group, 1 g of TXA was diluted in 50 ml of normal saline, injected into the surgical site (posterior and anterior capsule, medial and lateral retinaculum), and the surgical site was soaked in the solution for 5 min before deflation of the tourniquet. Treatment dose was determined from previous reports that indicate TXA 10–20 mg/kg IV and 1.0–3.0 g applied topically is effective for reducing blood loss in primary TKA [18–22]. Patients and investigators responsible for evaluating the results were blind to the treatment allocation, while two authors of the study were unblinded on the day of surgery.

All operations were carried out by the same surgeon. Patients’ limbs were elevated, bleeding was controlled with an Esmarch bandage, and the tourniquet was inflated to 350 mmHg. Operative techniques included a median skin incision followed by a joint incision. Gentamicin cement was used for prosthetic fixation. A polyethylene liner was inserted and wounds were closed. An intra-articular drain was used until post-operative day 2. All patients were given low-molecular-weight heparin to prevent DVT unless they took another cardiovascular medication before surgery.

Indications for blood transfusion were hemoglobin < 8.0 g/dl or hemoglobin < 10.0 g/dl with concomitant symptoms of anemia (tachycardia, tachypnea, decreased exercise tolerance) or anemia-related organ dysfunction.

All subjects underwent the same postoperative recovery and rehabilitation program. Routine follow-up was performed at postoperative 2, 6, and 12 weeks, and imaging analysis was performed at postoperative 6 weeks.

### Outcomes

The primary outcome measure was the difference in pre-operative and postoperative hemoglobin levels. Secondary outcome measures include intra-operative, post-operative, and total blood loss, number of transfusions and number of transfused units, post-operative VAS score for knee pain, and complications.

Hemoglobin was measured before the operation and at 24 h, 48 h, and 96 h post-operatively. The difference in pre-operative and postoperative hemoglobin levels was calculated. The postoperative hemoglobin level was defined as the lowest level of hemoglobin among three levels measured postoperatively. The amount of postoperative bleeding was estimated from the drainage volume. The VAS score for knee pain was measured using a 10 cm visual analog scale.

### Statistical analyses

Statistical analyses were conducted using IBM SPSS 12.0 for Windows (Microsoft Corporation, Redmond WA). Data are expressed as mean ± standard deviations (SDs). Continuous variables are presented as means and standard deviations. Categorical variables are summarized as the number and percentage of the total study population. Normally distributed continuous variables were compared with a two-sided independent t-test. Categorical variables were combined with Chi-square. Statistical significance was defined as *P* < 0.05.

## Results

This prospective randomized clinical trial included 64 patients undergoing TKA due to degenerative arthritis of the knee. Patients’ baseline demographic and clinical characteristics are shown in Table 1**.** Mean age of patients in the Intravenous Group was 66.47 years (range, 56–79 years); mean age of patients in the Topical Group was 66.43 years (range, 54–78 years). There were no significant differences in age, gender, height, weight, body mass index, prothrombin time, hemoglobin, American Society of Anesthesiologists score, or comorbidities between the two groups.

**Table 1:** Patient baseline demographic and clinical characteristics

	IV group	Topical group	*P* value
Age (year)	66.47 ± 8.28	66.43 ± 7.69	0.984
Male: Female	14:18	16:16	0.25
Body mass index (kg/m2)	32.39 ± 3.73	34.15 ± 5.02	0.084
American Society of Anesthesiologists classification	2.03 ± 0.14	1.79 ± 0.26	0.153
hemoglobin (g/l)	11.26 ± 1.60	10.97 ± 1.19	0.21
Red blood cell(10^12^/l)	3.72 ± 0.75	4.05 ± 1.04	0.35
prothrombin time(s)	11.51 ± 3.51	11.85 ± 4.62	0.16

There were no significant differences in intra-operative blood loss (intravenous: 122.81 ± 41.60 mL [range 60–200 mL] vs. topical: 109.06 ± 33.38 mL [range 70–200 mL]), post-operative blood loss (intravenous: 125.31 ± 35.649 mL [range 80–240 mL] vs. topical: 110.00 ± 30.900 mL [range 20–180 mL]), or total blood loss (intravenous: 254.38 ± 52.544 mL [range 140–420 mL] vs. topical: 210.00 ± 41.426 mL [range 100–400 mL]) in the Intravenous Group versus the Topical Group.

There were no significant differences in post-operative drop in hemoglobin (intravenous: 2.84 ± 0.68 [range 1.95–4.36] vs. topical: 2.66 ± 0.60 [range 1.73–3.84]). However, the number of transfused red blood cell units was significantly greater in the Intravenous Group (42 units red cell suspension; 1.28 units/patient) compared to the Topical Group (20 units red cell suspension; 0.63 units/patient) (*P* < 0.05) (Table 2).

**Table 2:** Outcomes

	IV group	Topical group	*P* value
Post-Operative Transfusion (Units)	1.28	0.63	0.016
Intra-operative blood loss (ml)	122.81 ± 41.60 mL	109.06 ± 33.38 ml	0.150
Post-operative blood loss(ml)	125.31 ± 35.649 mL	110.00 ± 30.900 mL	0.071
Total blood loss	254.38 ± 52.544 mL	210.00 ± 41.426 mL	0.143
Hb Fall (mg/dl)	2.84 ± 0.68	2.66 ± 0.60	0.261
VAS			
12 h	5.41 ± 0.875	6.69 ± 0.998	0.001
24 h	3.88 ± 0.976	5.50 ± 0.842	0.008
48 h	2.75 ± 0.568	2.97 ± 0.647	0.157
comorbidities	no	no	

Post-operative VAS score was significantly lower in the Intravenous Group compared to the Topical Group at 12 and 24 h after surgery. In the Intravenous Group, the VAS score was 5.41 ± 0.875 at post-operative 12 h and 3.88 ± 0.976 at post-operative 24 h. In the Topical Group, the VAS score was 6.69 ± 0.998 at post-operative 12 h and 5.50 ± 0.842 at post-operative 24 h. At 48 h after surgery, there was no significant difference in VAS score (intravenous: 2.75 ± 0.568 vs. topical: 2.97 ± 0.647).

There were no differences in post-operative thromboembolic complications between the Intravenous Group and Topical Group. No infusion-related complications related to TXA dosing, no allergic reactions, and no deaths were reported.

## Discussion

This study sought to compare the clinical effectiveness of IV and topical TXA in primary TKA using a prospective randomized controlled trial. Our results indicate that topical TXA and IV-TXA have similar safety and efficacy for reducing perioperative blood loss in TKA. There was no significant difference in the change in hemoglobin levels or therapeutic effect between topical TXA and IV administration. However, patients reported a significantly higher VAS score for knee pain in the Topical Group compared to the Intravenous Group at 12 and 24 h after surgery; there was no significant difference at 48 h. The higher VAS score at 12–24 h after the surgery may result from increased pressure in the knee joint due to the topical administration of TXA. A previous study [22] found that topical administration of TXA via a drain tube significantly increased joint swelling following TKA; rehabilitation in these patients took longer because of the pain. Further studies are warranted to elucidate the clinical and economic implications of the increased VAS score in patients receiving topical administration of TXA in primary TKA.

The current study confirmed the results of previous studies showing that IV and topical TXA have similar safety and efficacy profiles in TKA [12–16]. However, to the authors’ knowledge, this is the first study to include patient-reported outcomes in a study investigating the effectiveness of different regimens of TXA administration. Results showed that IV TXA administration may be the optimal approach as patients reported less pain during the initial 24 h after surgery, suggesting this regimen is associated with the potential for faster rehabilitation and shorter length of hospital stay.

TKA has good outcomes for the treatment of end-stage knee arthritis; however, blood loss may vary from 800 ml to 1800 ml [1–5], and the incidence of transfusion may range from 11 to 67% [6, 7]. Allogeneic blood transfusions are an economic burden and may result in the transmission of bloodborne pathogens, an immunomodulatory response, and periprosthetic joint infection [23]. Control of perioperative blood loss is associated with lower transfusion rates and shorter length of hospital stay [24, 25]. Currently, perioperative blood loss is controlled by the use of a tourniquet, hypotension, re-infusion drains, bipolar radiofrequency ablation, and antifibrinolytic drugs such as TXA.

TXA is an anti fibrinolytic drug that is used to reduce hemorrhage. TXA enhances microvascular hemostasis by preventing the dissolution of fibrin clots [26]. TXA is rapidly distributed to the synovial fluid and synovial membranes. The biological half-life of TXA in synovial fluid is estimated at 3 h [27]. TXA is eliminated through glomerular filtration; excretion of an Intravenous infusion of 10 mg / kg may reach 30% at 1 h, 55% at 3 h, and 90% at 24 h [27]. Methods of TXA administration include intravenous or Intramuscular injection or oral, [28], which result in maximal plasma levels of TXA at 5 to 15 min, 30 min, or2 hours, respectively [29, 30]. TXA may also be administered by intra-articular injection.

Renal insufficiency, previous history of DVT, and cerebrovascular and heart disease are contraindications to the use of IV TXA. In these patients, topical application of TXA to the knee joint is considered the safest route of administration as drug activity is delivered directly to the site of action and systemic sequelae are limited [31]. To target the bleeding, TXA is applied locally before wound closure. This reduces the significant increase in local fibrinolysis associated with tourniquet release [32]. Akizuki first described the use of topical TXA in orthopedic surgery [33]. They found that intra-articular application of 250 mg TXA in 50 ml saline effectively reduced bleeding in patients with cementless TKA. Since then, other studies have demonstrated similar outcomes. A prospective, double-blind, placebo-controlled randomized trial of 124 patients who received 1.5 g or 3 g of TXA or placebo in 100 ml of saline in the joint after surgery showed that TXA administration resulted in a significantly lower drop in hemoglobin loss and blood loss with no differences in thromboembolic complications. In accordance with our findings, a prospective randomized study involving 89 patients showed no significant difference in lowest post-operative hemoglobin level or total drain output following topical administration of 2.0 g TXA or 10 mg/kg TXA IV [34].

The authors acknowledge that the current study has several limitations. First, the study was conducted in a small sample of patients. Second, the clinical characteristics of thromboembolic complications were not documented. A longer follow-up period is required to adequately compare the safety profile and functional outcome differences between the two groups.

## Conclusion

This study showed that topical TXA is not inferior to IV administration in reducing perioperative blood loss in primary TKA. However, the VAS score in the Topical Group was significantly higher than the Intravenous Group at 12 and 24 h after surgery. The influence of the injection volume of locally applied TXA on post-operative pain in surgical patients warrants further investigation.

## Abbreviations

DVT: Deep vein thrombosis
IV: Intravenous
TKA: Total knee arthroplasty
TXA: Tranexamic acid
VAS: Visual Analog Scale
